# The de Morton Mobility Index (DEMMI): An essential health index for an ageing world

**DOI:** 10.1186/1477-7525-6-63

**Published:** 2008-08-19

**Authors:** Natalie A de Morton, Megan Davidson, Jennifer L Keating

**Affiliations:** 1 Department of Physiotherapy, School of Primary Health Care, Faculty of Medicine, Nursing and Health Sciences, Monash University – Peninsula Campus, PO Box 527, Frankston, Victoria, 3199, Australia; 2 The Northern Clinical Research Center, Northern Health, 185 Cooper St, Epping, Victoria, 3076, Australia; 3 School of Physiotherapy, Division of Allied Health, Faculty of Health Sciences, La Trobe University, Victoria, 3086, Australia

## Abstract

**Background:**

Existing instruments for measuring mobility are inadequate for accurately assessing older people across the broad spectrum of abilities. Like other indices that monitor critical aspects of health such as blood pressure tests, a mobility test for all older acute medical patients provides essential health data. We have developed and validated an instrument that captures essential information about the mobility status of older acute medical patients.

**Methods:**

Items suitable for a new mobility instrument were generated from existing scales, patient interviews and focus groups with experts. 51 items were pilot tested on older acute medical inpatients. An interval-level unidimensional mobility measure was constructed using Rasch analysis. The final item set required minimal equipment and was quick and simple to administer. The *de Morton Mobility Index *(DEMMI) was validated on an independent sample of older acute medical inpatients and its clinimetric properties confirmed.

**Results:**

The DEMMI is a 15 item unidimensional measure of mobility. Reliability (MDC_90_), validity and the minimally clinically important difference (MCID) of the DEMMI were consistent across independent samples. The MDC_90 _and MCID were 9 and 10 points respectively (on the 100 point Rasch converted interval DEMMI scale).

**Conclusion:**

The DEMMI provides clinicians and researchers with a valid interval-level method for accurately measuring and monitoring mobility levels of older acute medical patients. DEMMI validation studies are underway in other clinical settings and in the community. Given the ageing population and the importance of mobility for health and community participation, there has never been a greater need for this instrument.

## Background

Contemporary beliefs are that physical decline is not the natural partner of aging and that people can remain physically able and independent for the duration of their lives. This progressive position is reflected in encouragement of regular exercise and activity in older people [1,2]. However, by systematically reviewing existing instruments, we identified that a broadly applicable instrument that accurately measures and monitors mobility of older adults across the spectrum of health does not exist [3]. In this systematic review, the Elderly Mobility Scale (EMS) [4], Hierarchical Assessment of Balance and Mobility (HABAM) [5] and the Physical Performance Mobility Examination (PPME) [6] were identified as potentially suitable. However, clinimetric evaluation indicated significant limitations with each of these mobility instruments.

The HABAM, EMS and PPME were each designed for measuring the mobility of hospitalised older patients. Following clinimetric evaluation [3], the HABAM was identified to have the most desirable properties of these existing instruments. However, an important limitation of the HABAM is a ceiling effect (25% of persons scoring the highest possible score) in an older acute medical population [5]. These findings support the proposal that a new mobility instrument is required for older acute medical patients.

Two common instruments for assessing mobility in the acute hospital environment are the Timed Up and Go test (TUG) [7] and the Barthel Index (BI)[8]. However, these instruments have inadequate scale width [9-13] to capture changes in physical health for people whose limitations are either severe or relatively modest. The TUG has a floor effect with approximately one quarter of patients unable to complete this test because they are too weak [10] and the BI has a ceiling effect with approximately one quarter of patients scoring within the error margin of the highest score [10].

Mobility is an important indicator of the health status of older people. According to the World Health Organisation's International Classification of Functioning (ICF) [14] 'mobility' is classified as one of nine domains of 'activity and participation' and is defined as "moving by changing body position or location or by transferring from one place to another, by carrying moving or manipulating objects, by walking, running or climbing, and by using various forms of transportation."

Without an accurate mobility instrument, healthcare providers cannot accurately monitor deterioration in mobility and appropriate strategies to maintain physical health may not be triggered. It has been argued that inadequate measures of physical ability, across the spectrum of abilities that exist in older people, presents the most pressing issue in exercise gerontology [15]. It has also been suggested that until such measures exist, our understanding of particular aspects of physical ageing will be limited [16].

Hospitalised people have a diverse range of acute clinical presentations and co-morbid conditions. The primary aim of this research was to develop a practical and high quality instrument with the scale width for measuring the mobility status of all hospitalised older medical patients. A fundamental aspect of instrument design was that data would be based on observation of performance rather than patient or proxy recall of mobility to avoid distortion associated with poor recall or cognitive deficits [17].

## Methods

The four phases in instrument development were approved by the Ethics Committees at The Northern Hospital and/or Monash University.

### Phase 1: Item generation and development

Items were generated from existing mobility scales, 3 focus groups with academics and clinicians from relevant healthcare disciplines (*n *= 24) and patient interviews (*n *= 12). Items were sought that assessed older people across the spectrum of mobility from bed bound to fully active and the search for relevant items continued to the point where additional information became redundant. Two independent assessors applied pre-determined criteria. To be included, it was necessary that the item

• was able to be easily administered i.e. can be performed at the patient's bedside

• was brief to conduct

• was administered based on observation of patient performance

• could be administered by professionals from different healthcare professions

• was appropriate to administer in an acute care hospital

• could be safely administered to patients who have an acute medical condition

• required minimal equipment

• provided measurable information about patient mobility

• provided objective information about patient mobility that would facilitate goal setting

for treatment

• administration could be clearly and unambiguously defined

• provided information that was not duplicated by another item

Using consensus of experts, unambiguous and practical testing protocols were developed for 51 mobility items that remained after two independent assessors removed redundant items and applied inclusion criteria.

### Phase 2: Item testing

#### Participants

Participants were recruited from general medical wards at The Northern Hospital, Victoria, Australia. Consecutive participants were screened by a recruiting officer and were eligible to participate if 65 years or older and were assessed within 48 hours of admission. Patients were excluded if they had a planned hospital stay of less than 48 hours, severe dysphasia, documented contra-indications to mobilization, were isolated for infection, or if death was imminent. All eligible participants were invited to participate. Consent was obtained within 48 hours of hospital admission. For patients deemed incompetent to consent, this was obtained from the 'person responsible' or next-of-kin. Interpreters were employed when required.

#### Testing procedure

Participants were assessed at the bedside every 48 hours during hospitalisation or on the Monday following a weekend. Baseline measurements included age, sex, place of residence prior to admission, primary language, gait aid use prior to hospitalisation, Mini Mental State Examination (MMSE) [18], Charlson Comorbidity Index [19], APACHE11 Severity of Illness Scale [20], the Barthel Index (BI) [8,21], Hierarchical Assessment of Balance and Mobility (HABAM) [5] and the new mobility items. The BI and HABAM were selected for a head-to-head comparison with the new mobility instrument. The BI is widely used as a self report measure of independence in activities of daily living in the acute hospital setting [11] and, prior to this study, the HABAM was identified as having the most desirable properties of existing mobility instruments [3]. Each of these outcome measures are described in further detail below.

At each assessment a researcher administered the BI and the MMSE. As close as possible to this assessment, the patient was assessed on the mobility items by the principal researcher, who was blind to BI scores. The HABAM items were a subset of these mobility items.

Mobility items were administered in the order of bed, chair, balance and walking activities to maximise patient safety, confidence and ease of testing. Familiarisation trials were not provided to minimise fatigue and time required to administer the test. At each test the therapist and patient independently rated the patient's current mobility compared with admission mobility on a 5 point scale (much worse, bit worse, same, bit better, much better). This provided a reference standard for important change in mobility.

#### Outcome measures

The APACHE 11 is a severity of illness scale with a score range from 0 to 71, where higher scores represent increasing severity of illness during the first 24 hours of hospital admission. The Charlson Index classifies comorbid conditions according to risk of mortality. One year mortality rates in a medical population have been reported to be 12%, 26%, 52% and 85% for Charlson scores of 0, 1–2, 3–4 and greater than 5 respectively [19].

The modified BI is an ordinal scale that provides a total score between 0 and 100 where higher scores indicate greater independence in activities of daily living [21]. The HABAM is an interval level mobility instrument that provides a score between 0 and 26 [5] where higher scores indicate increasing levels of independent mobility and was designed for application in an older acute medical population. The MMSE is reported to be a valid and reliable measure of patient cognition [18]. It provides a score between 0 and 30 points where increasing scores indicate better cognitive ability.

#### Item reduction

The complete set of 51 mobility items were pilot tested for two weeks to remove items with practical limitations, a process that included patient and assessor interview about the mobility tests. The remaining 42 items were then tested on a large sample by the principal researcher. After completion, items with practical limitations were removed and Rasch analysis conducted.

#### Rasch analysis

Data analyses were performed using SPSS version 12.0 [22] and RUMM2020 [23]. The Rasch partial credit model was employed to identify misfitting and redundant items and to identify a hierarchy of mobility items ranked from easiest to hardest. Participants were divided into 3 class intervals (ie, 3 groups of patients at different levels of mobility). Item misfit was considered if the chi-square or F statistic probability value was less than the Bonferroni-adjusted a value for multiple testing or the fit residuals were greater than ± 2.

Item residuals from Rasch analysis were also examined as a finding of no association between residuals for individual items has been argued as evidence of local item independence [24]. High positive correlation between residuals provides evidence of local item dependence and high negative correlations is thought to indicate multidimensionality.

Differential item functioning (DIF) analysis [25] was planned for age, gender, time of assessment, cognitive status (MMSE) and whether an interpreter was required. DIF was considered significant if the chi-square probability value was lower than the Bonferroni-adjusted *p *value. *A priori*, these factors were considered potential confounders to item functioning.

Item response thresholds were also studied to investigate the existence of disordered thresholds, that is, response patterns on the rating scale that are not in the expected order. The person separation index (PSI) was reported to provide an indication of the internal consistency (reliability) of the scale by examining the ability of the instrument to discriminate among respondents.

Sample size for Rasch analysis was based on recommendations by Linacre et al [26]. These authors recommend a sample size of 64 – 144 to provide 95% confidence +/- 0.5 logits. Baseline and 48 hour assessments during a 3–4 month period were expected to provide more than 200 assessments. In the absence of DIF by time, all available assessments would be included for Rasch analysis as recommended by Wright [27] and Chang and Chang [28].

### Phase 3: Interval scoring system and clinimetric evaluation (development sample)

Based on Rasch analysis, an interval scoring system (0–100) was developed to facilitate clinical application and clinimetric evaluation of the reduced item set.

#### Reliability study

An inter-rater reliability study was conducted on a subset of patients who reported no fatigue from the first assessment. After the first assessment and a 10 minute rest, the mobility assessment was repeated by a physiotherapist blind to the outcomes of the first test. Test order of assessing physiotherapists was randomised. Power calculations were performed based on recommendations by Walter et al [29]. The Minimal Detectable Change at 90% confidence (MDC_90_) and accompanying 95% confidence intervals were estimated [30].

#### Validity

Correlation coefficients and associated 95% confidence intervals were calculated to investigate the convergent validity of DEMMI scores with the BI (a measure of a related construct) and HABAM (a measure of the same construct), and discriminant validity with the MMSE, Charlson Index and APACHE 11 (measures of different constructs). To investigate known-groups validity, an independent t test was performed on DEMMI scores of patients discharged to home compared to inpatient rehabilitation.

#### Minimum clinically important difference

The MCID was calculated for DEMMI, HABAM and BI as the mean change score for patients who rated themselves 'much better' at discharge (criterion based method). The MCID was also calculated using distribution based method recommended by Norman et al[31].

#### Responsiveness to change

The Effect Size Index (distribution method)(ESI) and Guyatt's Responsiveness Index (criterion method)(GRI), were selected *a priori *to calculate measurement responsiveness of the DEMMI, HABAM and BI. Inferential 95% confidence bands were calculated to enable statistical comparison of responsiveness estimates as recommended by Tryon [32].

#### Time to administer

The time required to administer the DEMMI was rounded to the nearest 30 seconds and was recorded using a stop watch.

### Phase 4: Final DEMMI refinement and validation in an independent sample

Prior to testing in an independent sample, the DEMMI was administered by clinicians from several health care disciplines. Clinician responses to a set of structured, one-on-one interview questions were used to refine the instrument format, items and testing protocol.

The refined instrument was then tested on an independent sample of older acute medical patients and evaluated, as per phases 2 and 3. An independent physiotherapist (not involved in the instrument development) conducted the mobility assessments.

## Results

**Figure 1:**
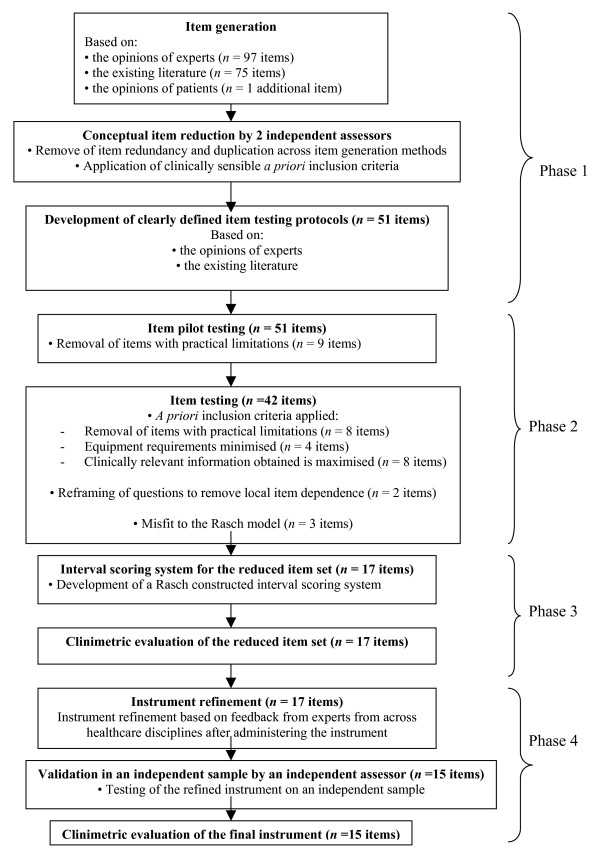
Stages of unidimensional instrument development.

The stages of instrument development in this study are summarised in Figure 1.

### Phase 1: Item generation and development

Ninety seven mobility items were generated from focus groups and 75 items from existing mobility instruments. One additional item was generated from patient interviews. After removal based on item duplication, redundancy and application of inclusion criteria, 51 items remained for pilot testing (Table 1).

**Table 1:** Reasons for item exclusion at each stage of instrument development

**Excluded item**	**Reason for exclusion**
**Pilot testing of 51 mobility items: 9 items excluded due to practical limitations**	

Number of times in/out of bed in 10 sec	Removed to maximise patient safety. Difficult to test for patients who have drips, drains, indwelling catheters etc. A similar item, 'lying to sitting independently within 10 seconds' was deemed to be safer and provided similar clinical information.
Sit to stand 3 times in 10 seconds	To reduce the burden of testing by minimising redundancy of sit to stand items. 'Independent sit to stand in 3 seconds' was retained due to shorter administration time.
Sitting balance and turning head	Many patients had significantly limited cervical range of movement and therefore this test was difficult to standardise across patients.
Reach sideways to pick up pen from floor (sitting)	Several patients reported feeling dizzy performing this task after first attempting to reach forward to pick up pen from floor. Reaching forwards to pick up a pen was considered to be the more functional item and was therefore retained.
Reach sideways to pick up pen from floor (standing)	As above
Walk 6 meters in 10 seconds	Requires a standardised walking test environment which could not be relied upon.
Step test	Requires a standardised step. Removed due to equipment requirements.
Step	Requires a standardised step. Removed due to equipment requirements.
Step over box	Requires a standardised step. Removed due to equipment requirements.

**Testing of 42 mobility items: 8 items excluded due to practical limitations**	

Skipping	This is a complex movement that required practice to perform in a standardised way.
Sit to stand using the chair seat (not using the arms of the chair)	For wider patients there was not enough space to push up from the seat. Cognitively impaired patients found this task difficult to understand when the arms of the chair were accessible.
Immediate standing balance	Required significant explanation, particularly for cognitively impaired patients.
Semi tandem stance	Required significant explanation and/or demonstration for patients to understand task.
Reach in sitting	Dizziness prevented some patients from successfully completing this item.
360 degree turn	This item was difficult to perform with patients who had lines, drips, drains etc.
Sit to lie	Asking the patient to return to bed to assess this item interrupted the flow of testing.
Hop	This is a dynamic single leg activity and was removed to maximise patient safety.

**Reframing walking items to remove potential for local item dependence (assumption of Rasch analysis)**	

Four walking items: 5 m, 10 m, 20 m and 50 m (response options were levels of assistance for each distance)	4 walking items replaced with 2 items:
	1. walks +/- gait aid (with distance response options)
	2. walking assistance (with levels of assistance for response options)

**Rasch analysis of 32 mobility items: 4 items removed**	

Transferring from bed to chair	Required equipment and had similar threshold locations to other items
Carrying a glass of water while walking	Required equipment and had similar threshold locations to other items
Timed bed transfer	Required equipment and had similar threshold locations to other items
Timed chair transfer	Required equipment and had similar threshold locations to other items

**Removal of items that provided similar clinical information (and to avoid local item dependence): 8 items removed**	

Sitting arm raise	Similar items: Sitting unsupported and sitting arm raise
	'Sitting unsupported' is a simpler test and maximises scale width as it has the lowest logit item score (easiest item).
×5 sit to stand without arms	Similar items: ×1 sit to stand without arms and ×5 sit to stand without arms. 'x1 sit to stand without arms' is a simpler and quicker test.
Standing arm raise Standing with eyes closed	Similar items: Standing unsupported, standing arm raise and standing with eyes closed. 'Standing unsupported' is the simplest test and is an important component of independent mobility.
Standing with feet together eyes closed	Similar items: Standing with feet together and standing with feet together eyes closed
	'Standing with feet together' is a simpler test.
Tandem standing Tandem walking	Similar items: Tandem standing, tandem standing with eyes closed and tandem walking
	'Tandem standing with eyes closed' had the second highest item logit location (second most difficult item) and was therefore retained to maximise scale width.
Stand on one leg	Similar items: Stand on one leg and stand on one leg eyes closed
	'Stand on one leg with eyes closed' had the highest item logit location (most difficult item) and was therefore retained to maximise scale width.

**Rasch analysis of 20 mobility items: 3 items removed**	

Toe walk	Similar threshold locations to other items and statistically significant misfit
Heel walk	Similar threshold locations to other items and statistically significant misfit
Sideways walking	Similar threshold locations to other items and statistically significant misfit

### Phase 2: Item testing

#### Pilot testing 51 mobility items

Pilot testing on 15 consecutive older general medical patients identified 9 items for removal based on practical limitations (Table 1).

#### Testing of 42 remaining mobility items

**Figure 2:**
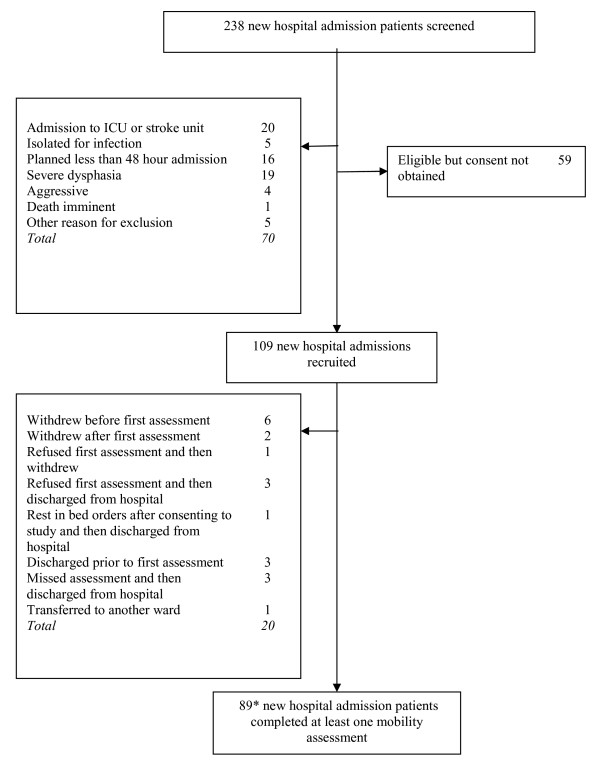
**Development sample: flow of participants through the study**. *3 patients were readmitted during the study period and were tested twice as 'new admissions.'

Figure 2 shows that of the 388 new hospital admissions screened for inclusion, 219 were eligible, 104 were recruited and 89 performed at least one mobility assessment. Three patients were readmitted during the study period and were included twice as new hospital admissions. Table 2 shows the admission characteristics for the 86 patients included in this study. There were no adverse events as a result of the mobility assessments. A further 8 items were removed due to practical limitations that were identified following further testing and the 4 walking items were rescored to 2 items to limit local item dependence (an assumption of Rasch analysis)(Table 1).

**Table 2:** Patient baseline demographics for the instrument development and validation

**Patient Baseline demographics**	**Development study *n *= 86**	**Validation study *n *= 106**
**Mean Age years (sd)**	79.2 (7.1)	81.2 (7.3)
**Gender (% female)**	53%	47.3%
**Place of prior residence**		
Home alone	24 (27.9%)	31 (29.3%)
Home accompanied	52 (60.5%)	65 (61.3%)
Hostel/SRS	6 (7%)	8 (7.6%)
Nursing Home	4 (4.7%)	2 (1.9%)
**Primary Language**		
English	59 (68.6%)	75 (69.8%)
Italian	17 (19.8%)	14 (13.2%)
Macedonian	3 (3.5%)	1 (0.9%)
Other	7 (8.1%)	17 (16.1%)
**Gait aid prior to hospital admission**		
None	32 (37.2%)	50 (44.6%)
Walking stick	26 (30.2%)	22 (19.6%)
Frame	25 (29.1%)	37 (33%)
Other	3 (3.5%)	3 (2.7%)
**Primary Diagnosis**		
Circulatory	20 (23.3%)	21 (19.8%)
Respiratory	13 (15.1%)	37 (34.9%)
Endocrine	9 (10.5%)	6 (5.7%)
Digestive	4 (4.7%)	7 (6.6%)
Genitourinary	4 (4.7%)	6 (5.7%)
Musculoskeletal	4 (4.7%)	3 (2.8%)
Other	32 (37.2%)	26 (24.5%)
**Mean Charlson Index (sd)**	1.83 (1.54), *n *= 84	1.94 (1.57), *n *= 105
**Mean APACHE II (sd)**	11.89 (3.10), *n *= 83	12.60 (3.77), *n *= 105
**Mean MMSE (sd)**	21.73 (7.57), *range 0–30 n *= 85	22.77 (6.30), range 1–30*, n *= 103
**Mean Barthel Index (sd)**	81.29 (22.72), range 20–100	82.47 (18.80), range 15–100, *n *= 105
**Mean HABAM (sd)**	18.06 (6.78), range 0–26	16.83 (6.77), range 0–26

#### Rasch analysis of 32 mobility items

Following item testing and Rasch analysis, 32 items were reduced to 17 (Table 1). DIF by time was not identified for the 17 items and therefore Rasch analysis was performed on data from hospital admission and subsequent 48 hour assessments. Rescoring three items (*lie to sit, sit to stand and walking distance*) produced ordered thresholds for all items.

Data for the 17 mobility items fitted the Rasch model (item-trait χ^2 ^= 41.17, df = 34, p = 0.19). The t test procedure [24,33] identified that the percentage of individual t tests outside the acceptable range was only 4.23%. (95% CI 1.0% to 7.0%). This provides further evidence of the unidimensionality of the 17 mobility items.

Examination of the residual correlation matrix indicated negative correlations of greater than 0.3 between *sit unsupported *and *bridge *(r = -0.55), *standing on toes *and *stand on one leg eyes closed *(r = -0.58) and *tandem standing eyes closed *and *walking distance *(r = 0.35). However, these findings were not supported by high fit residuals for any of these items. A positive correlation of greater than 0.30 was only identified between the *roll *and *lie to sit *(r = +0.37) items. Although this result indicates the possibility of some response dependency between these mobility tasks, both items were retained as each provides important clinical information regarding patient mobility and care needs during acute hospitalisation. In addition, examination of the admission only dataset indicated a lower correlation of +0.21.

Person separation was 0.92, indicating the test could discriminate 5.8 strata of ability.

### Phase 3: Interval scoring system and clinimetric evaluation

Raw scores for the reduced item set were converted to a 0–100 interval scale. The clinimetric properties for the 17 item DEMMI are reported in Table 3.

**Table 3:** Clinimetric properties of the DEMMI

**Clinimetric property**	**Development study 17 items**	**Validation study 15 items**
**Reliability, MDC**_90_**(95%CI)**		
*Inter rater*	9.5 (5.0 to 13.3), *n *= 21	8.90 (6.3 to 12.7), *n *= 35
		
**MCID (95%CI)**		
*Criterion based method*	7.8 (5.3 to 10.2)	9.43 (5.9 to 12.9)
*Distribution based method*	8.0	10.5
		
**Construct Validity (r, 95%CI)**		
Convergent		
*HABAM*	0.92 (0.88 to 0.95), p = 0.00	0.91 (0.87 to 0.94), p = 0.00
*Barthel Index*	0.76 (0.65 to 0.84), p = 0.00	0.68 (0.56 to 0.77), p = 0.00
Discriminant		
*MMSE*	0.36 (0.16 to 0.53), p = 0.00	0.24 (0.05 to 0.41), p = 0.02
*APACHE 11*	-0.11 (-0.32 to 0.11), p = 0.18	0.07 (-0.12 to 0.26), p = 0.49
*Charlson*	-0.19 (-0.39 to 0.03), p = 0.11	-0.04 (-0.23 to 0.15), p = 0.68
Known Groups (DEMMI, 95%CI)		
discharge to rehabilitation	37.54 (33.99 to 45.10), *n *= 11	50.75 (42.39 to 59.11)*n *= 8
discharge to home	59.61 (56.32 to 62.90), *n *= 62 Independent t test: p = 0.00	62.14 (57.80 to 66.49) *n *= 70 Independent t test: p = 0.03
		
**Responsiveness to change**^#^		
Effect Size Index^#^		
*DEMMI*	0.37 (0.28 to 0.46)	0.39 (0.28 to 0.50)*
*HABAM*	0.31 (0.20 to 0.43)	0.35 (0.23 to 0.47)
*Barthel Index*	0.30 (0.17 to 0.44)	0.13 (0.01 to 0.25)*
GRI (patient)^#^		
*DEMMI*	1.23 (0.90 to 1.56)	0.92 (0.66 to 1.17)*
*HABAM*	1.00 (0.46 to 1.55)	0.72 (0.49 to 0.94)
*Barthel Index*	0.48 (0.01 to 0.95)	0.43 (0.21 to 0.65)*
GRI (therapist)^#^		
*DEMMI*	2.06 (1.60 to 2.51)	1.73 (1.37 to 2.09)*
*HABAM*	2.62 (1.70 to 3.54)	1.17 (0.86 to 1.48)
*Barthel Index*	1.58 (0.56 to 2.60)	0.65 (0.37 to 0.93)*
**Floor effect**	0%	<1%
**Ceiling effect**	<1%	3.8%
**Time to administer, mean (sd)**	13 mins 42 seconds (4.99 mins) for 42 mobility items	8 mins 47 seconds (3.89 minutes) for 17 mobility items

GRI = Guyatt's Responsiveness Index, ^# ^Tryon's inferential confidence intervals

* significant difference: evidenced by non overlapping inferential confidence intervals

#### Reliability

Correlation between independent assessor DEMMI interval scores was high (Pearson's r = 0.94, 95% CI 0.86 to 0.98). The mean scores for assessors 1 and 2 were 57.19 (sd = 17.07) and 55.05 (sd = 13.77) points respectively. A paired t test indicated no systematic differences between assessors (p = 0.14). Using a pooled standard deviation of 15.51, the standard error of measurement (SEM) was 4.10 and the inter-rater reliability MDC_90 _was 9.51 points (95% CI 5.04 to 13.32) on the 100 point DEMMI interval scale. This indicates that a patient needs to improve or deteriorate by 10 points or more for a clinician to be 90% confident that a true change in patient condition has occurred. A paired t test indicated no systematic difference between the first and second assessment scores (p = 0.77).

#### Validity

DEMMI scores had a significant and high correlation with HABAM and BI scores. This provides evidence of convergent validity for the DEMMI.

Discriminant validity for the DEMMI was evidenced by a low correlation with measures of other constructs (MMSE, APACHE 11 severity of illness and Charlson co-morbidity index scores).

An independent t test showed that patients who were discharged to inpatient rehabilitation had significantly lower DEMMI scores at acute hospital discharge than those discharged to home. Patients discharged to inpatient rehabilitation had a mean DEMMI score of 39.55 (sd = 9.41, 95% CI 33.72 to 45.38) and patients discharged to home had a mean DEMMI score of 59.61 (sd = 13.22, 95% CI 56.30 to 62.93). This provides evidence of known groups validity for the DEMMI.

#### Responsiveness

There was no significant difference identified between the responsiveness of DEMMI and HABAM measurements or DEMMI and BI measurements using the ESI or GRI based on patient or therapist report of change.

#### Minimally clinically important difference

By calculating the average change in DEMMI score for patients who reported to be 'much better' in their mobility between hospital admission and discharge, the MCID for the DEMMI was identified to be 8 points, that is, a change of 8 points or more is likely to represent a patient perceived important change in mobility. Using Norman et al.'s [31] distribution based method, the MCID was also calculated to be 8 points for the DEMMI.

### Phase 4: Final DEMMI refinement and validation in an independent sample

#### Item refinement

Feedback from 15 clinicians was obtained following their administration of the DEMMI. Minor changes were made to the *sit unsupported *item and testing protocol and the final format of the DEMMI.

#### Validation in an independent sample

**Figure 3:**
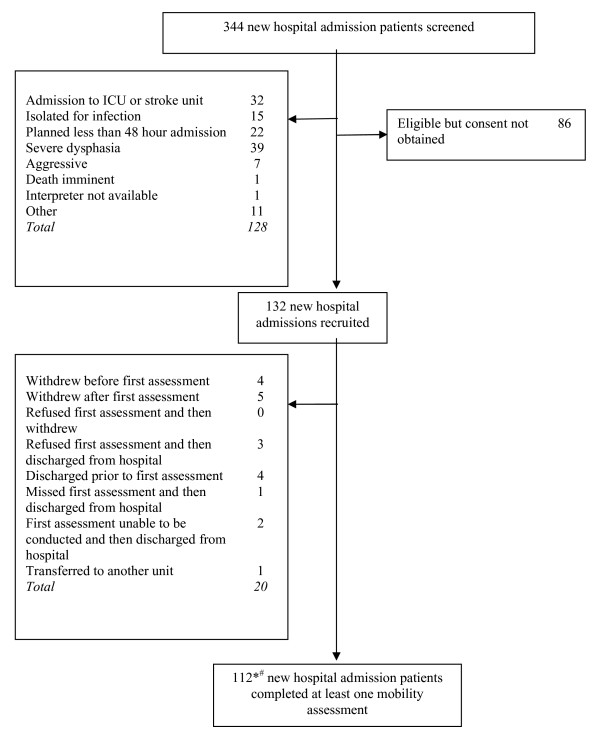
**Validation sample: flow of participants through the study**. * 6 patients were readmitted during the study period and were tested twice as 'new admissions.' # 106 'new admission' patients (100 patients) completed a hospital admission assessment (6 patients did not perform an admission assessment)

Figure 3 shows that of 344 new hospital admissions screened, 216 were eligible, 132 were recruited and 112 performed at least one mobility assessment. Six patients were readmitted during the study period and were included twice as new hospital admissions. Another six patients did not complete a hospital admission assessment. Table 2 shows the admission characteristics for the 106 patients included in this study. A total of 312 mobility assessments were performed using the 17 mobility items. Patients in the validation study did not differ from the instrument development sample on any baseline characteristic.

Prior to conducting Rasch analysis the *jog *item was removed. This item required clinical experience of medical conditions to determine whether testing should proceed. No participant was able to successfully complete the *standing on one leg with eyes closed *item in the validation study. Rasch analysis was therefore performed for the remaining 15 items.

In the validation study, the pooled dataset showed misfit to the Rasch model due to large sample size as there was no evidence of DIF by time or multidimensionality. Using the t test procedure [24,33], multidimensionality was not identified. Four items *(reaching for pen, backward walking, standing on toes and sit to stand no arms) *had a positive correlation of 0.3 or greater and three items *(walking distance, roll and lie-sit) *had a negative correlation of 0.3 or greater with the first residual component. The t test procedure indicated the percentage of individual t tests outside the acceptable range was 4.88% (95% CI -2.0% to 7.0%). This provides further evidence of the unidimensionality of the 15 DEMMI items and therefore does not explain the misfit of the data to the Rasch model. No evidence of local item dependency was identified as there was an absence of correlations in the residuals above a magnitude of 0.3.

Data fitted the model at each assessment time point; baseline (χ^2 ^= 24.60, df = 30, p = 0.74), first 48 hour assessment (χ^2 ^= 36.37, df = 30, p = 0.20) and subsequent 48 hour assessments (χ^2 ^= 36.26, df = 28, p = 0.14). Given the similar findings across samples, analysis of hospital admission data is reported.

**Figure 4:**
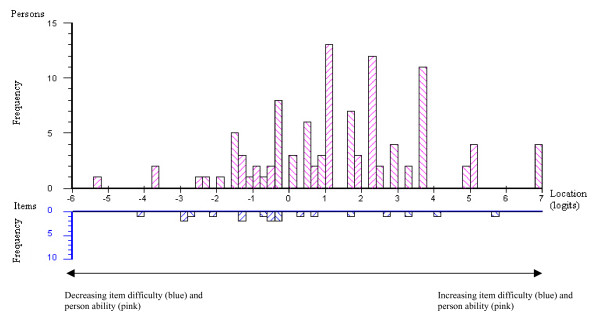
Person-item threshold graph for admission mobility assessments for the 15 item DEMMI in the validation sample.

There were 106 hospital admission mobility assessments performed. The mobility items were well targeted for older acute medical patients. Figure 4 shows the average item difficulty and the person ability locations on the logit scale. There were only a few persons with a lower ability than the easiest item (to the left of the scale), or with a higher ability than the hardest item (to the right of the scale). None of the items showed misfit to the model and χ^2 ^and F statistic Bonferroni adjusted probability values for the 15 items were non significant. Person separation was 0.88, indicating the test could reliably identify 3.7 strata of ability.

Three items showed significant DIF by age but appear to be statistical artifacts due to a small number of participants in one of the three class intervals. Two items (*lie to sit *and *walking independence*) showed mild disordering of one threshold. However, inspection of item thresholds in the pooled dataset showed these items to be ordered and they were not rescored.

**Figure 5:**
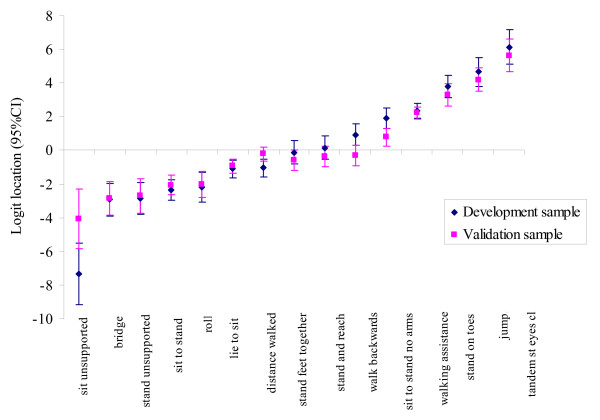
Item logit location for baseline data for the scale development and validation studies.

Figure 5 shows the item hierarchy for the 15 DEMMI items was consistent across independent samples. The final DEMMI is shown in Additional file 1 and its clinimetric properties reported in Table 3. The measurement properties of the DEMMI (Rasch, reliability, validity and MCID) were consistent with estimates obtained from the instrument development sample.

## Discussion

The DEMMI provides clinicians and researchers with an advanced, practical and reliable instrument for measuring mobility in hospitalised older acute medical patients. The DEMMI is a unidimensional instrument that measures mobility across the spectrum from bed bound to independent mobility. It is safe, quick and easy to administer, has minimal equipment requirements, can be administered at a patient's bedside and provides interval data.

The DEMMI overcomes ceiling effects identified in the BI and HABAM and the floor effect identified in the Timed Up and Go in an older acute medical patient population. The DEMMI items cover the broad spectrum of mobility levels that exist for older acute general medical patients as neither ceiling nor floor effect were identified. Therefore this instrument has the width required to measure improvement and deterioration in mobility across the spectrum of mobility levels that exist in an older acute medical patient population.

The DEMMI contains items that are considered to be important hallmarks of independent mobility and have face validity for measuring the domain of mobility as defined by the World Health Organisation [14]. Therefore this new mobility instrument facilitates the comprehensive assessment of mobility for older medical patients and assessment findings can be used to assist in goal setting for therapeutic intervention. For example, an older medical patient who has a logit location of -2.4 (or interval measure score of 38 at hospital admission) would be expected to be able to perform bed based mobility tasks, require minimal assistance or supervision for transfers in and out of the chair, have adequate balance to sit and stand unsupported and walk short distances with assistance or supervision. The mobility hierarchy indicates that for this patient to progress along the mobility continuum, goals for therapeutic intervention should include achieving independence in bed and chair transfers, then increasing walking distances and improving standing balance. Any item that the patient cannot do that they would be expected to be able to complete based on their total score can also be easily identified using this method (i.e. items lower in the mobility hierarchy than other items that were successfully completed).

The DEMMI has minimal equipment requirements and the scale protocol and scoring system fit onto one page (back and front). Only a bed or plinth, arm chair (seat height 45 cm) and pen are required to conduct the test. These pieces of equipment are usually readily available in hospital wards or emergency departments. The DEMMI can be quickly and easily applied in an acute hospital where the time and space available for testing would be similar, if not more constrained, than other clinical settings. Since the DEMMI is appropriate and without practical limitations for the broad spectrum of conditions seen in older acute medical patients, it is likely to also be safe for administration in most clinical populations.

The DEMMI item hierarchy was consistent across independent samples despite being administered by differing clinicians and testing a smaller number of items in the validation study. In support of earlier studies [5,34], this provides strong evidence that for heterogeneous older patient populations, physical recovery from illness follows a common path. Since older patients are expected to progress across the DEMMI mobility continuum in a predictable manner, the DEMMI hierarchy provides clinicians with a systematic method for identifying capabilities and limitations. The DEMMI facilitates comprehensive assessment of mobility and assessment findings can be used to define specific targets for therapeutic intervention.

Fit of the data to the Rasch model validates the summation of mobility item scores to produce a total mobility score and indicates that the DEMMI provides interval level data. A simple conversion table allows ordinal mobility scores (out of 19) to be converted to interval mobility scores (out of 100). For statistical purposes, a group mean increase of 20 (converted) points, for example, represents the same amount of improvement in mobility across individuals regardless of whether the patient is bed bound or independently mobile at initial assessment. Interval data allows researchers to interpret parametric statistical tests of DEMMI data.

In the validation study, two items were tested but removed from analysis. The *jog *item was removed to maximise the potential for the DEMMI to be used by clinicians with varying clinical experience and from different healthcare disciplines. Since this item was tested last, patient performance on this item did not influence performance on other items. The *standing on one leg eyes closed *item was the most difficult item in the instrument development sample. Since no participants were able to successfully complete the *standing on one leg eyes closed *item in the validation study, this extreme item could not be included in Rasch analysis. However, given that the properties of the 15 DEMMI items were consistent across independent samples despite the differing number of items tested, removal of this item (attempted by only 30% of patients) is unlikely to have influenced the estimated clinimetric properties of the final instrument.

The consistency of the DEMMI across independent samples provides confidence in the interpretation and clinical application of DEMMI scores. The MDC_90 _indicates that a minimum change score of 9 Rasch converted points on the DEMMI is required for 90% confidence that a true change in patient mobility has occurred and the MCID indicates a minimum change of 10 points is required to represent a clinically important change in patient mobility. These data were derived from inter-rater error estimates. Since inter-rater reliability estimates are typically larger than intra-rater, our calculations provide clinicians with conservative estimates of measurement error.

Although the DEMMI was developed for acutely hospitalised older patients, the potential applications of this instrument are broad. Due to its inclusive scale width, the DEMMI has the potential to be used in many clinical settings and subsequently enhance the continuity of care of older adults across providers, clinical settings and in the community. Further research is underway to validate the DEMMI across clinical settings and in the community and to translate the DEMMI into other languages.

It is possible that sampling bias may exist in the data reported in this research. Firstly, the requirement to obtain written and informed consent may have resulted in the inclusion of a healthier and less cognitively impaired cohort of patients compared to a typical older acute medical population. Secondly, data collection for both the development and validation studies was conducted at the same hospital site. However, since Rasch analysis assesses the consistency of item response patterns relative to the total score, sampling bias will not have influenced the fit of the data to the Rasch model in this research.

## Conclusion

The DEMMI has been rigorously developed and validated. More than 500 DEMMI assessments have been conducted and the Rasch, reliability, validity and MCID properties of the DEMMI were consistent across independent samples of older acute medical patients. Maximising the independence of older people is fundamental to prolonging health and quality of life and reducing dependence on limited healthcare resources.

## Competing interests

The authors declare that they have no competing interests.

## Authors' contributions

Nd conceived and designed the study, acquired the data, analysed and interpreted the data, wrote the manuscript and has given final approval of the version to be published. MD contributed to the analysis and interpretation of the data, has been involved in the drafting of the manuscript and given approval for the version to be published. JK contributed to the conception and design of the study, the analysis and interpretation of data, drafting of the manuscript and has given final approval of the version to be published.

This research was presented by Dr Natalie de Morton at the *World Physical Therapy Congress*, Vancouver, Canada, June 2007, the *Australian Physiotherapy Association Conference*, Cairns, Australia, October 2007 and the *Australian Association of Gerontology Conference (NSW region)*, Woolongong, Australia, April 2008.

## Supplementary Material

Additional file 1 The DEMMI. Click here for file
