# Supplementary material for: The de Morton Mobility Index (DEMMI): An essential health index for an ageing world
Source: Health Qual Life Outcomes. 2008 Aug 19;6:63. doi: 10.1186/1477-7525-6-63 (PMC2551589; doi:10.1186/1477-7525-6-63)
Supplement: Additional file 1 — The DEMMI. [file 1477-7525-6-63-S1.pdf]

de Morton Mobility Index (DEMMI)

	0	1	2
**Bed**			
1. Bridge	☐ unable	☐ able	
2. Roll onto side	☐ unable	☐ able	
3. Lying to sitting	☐ unable	☐ min assist<br>☐ supervision	☐ independent
**Chair**			
4. Sit unsupported in chair	☐ unable	☐ 10 sec	
5. Sit to stand from chair	☐ unable	☐ min assist<br>☐ supervision	☐ independent
6. Sit to stand without using arms	☐ unable	☐ able	
**Static balance (no gait aid)**			
7. Stand unsupported	☐ unable	☐ 10 sec	
8. Stand feet together	☐ unable	☐ 10 sec	
9. Stand on toes	☐ unable	☐ 10 sec	
10. Tandem stand with eyes closed	☐ unable	☐ 10 sec	
**Walking**			
11. Walking distance +/- gait aid<br>*Gait aid (circle): nil/frame/stick/other*	☐ unable<br>☐ 5m	☐ 10m<br>☐ 20m	☐ 50m
12. Walking independence	☐ unable<br>☐ min assist<br>☐ supervision	☐ independent with gait aid	☐ independent without gait aid
**Dynamic balance (no gait aid)**			
13. Pick up pen from floor	☐ unable	☐ able	
14. Walks 4 steps backwards	☐ unable	☐ able	
15. Jump	☐ unable	☐ able	
COLUMN TOTAL SCORE:			

RAW SCORE TOTAL
(sum of column total scores) /19

DEMMI SCORE
(MDC_{90} = 9 points; MCID = 10 points) /100

easiest

sit unsupported

bridge

stand unsupported

sit to stand

roll

lie to sit

standing feet together

pick up pen from floor

walks backwards

walking distance

sit to stand no arms

walking independence

jump

stand on toes

tandem stand eyes closed

hardest

Raw-DEMMI Score Conversion Table

Raw Score	0	1	2	3	4	5	6	7	8	9	10	11	12	13	14	15	16	17	18	19
DEMMI score	0	8	15	20	24	27	30	33	36	39	41	44	48	53	57	62	67	74	85	100

Comments:

Signature:____ Date:________________

ITEM INSTRUCTIONS

Bed

1. Person is lying supine and is asked to bend their knees and lift their bottom clear of the bed.
2. Person is lying supine and is asked to roll onto one side without external assistance.
3. Person is lying supine and is asked to sit up over the edge of the bed.

Chair

4. Person is asked to maintain sitting balance for 10 seconds while seated on the chair, without holding arm rests, slumping or swaying. Knees and feet are placed together and feet can be resting on the floor.
5. Person is asked to rise from sitting to standing using the arm rests of the chair.
6. Person is asked to stand with their arms crossed over their chest.

Static Balance

7. The person is asked if they can stand for 10 seconds without external support.
8. The person is asked if, for 10 seconds, they can stand with their feet together.
9. The person is asked if they can stand on their toes for 10 seconds.
10. The person is asked to place the heel of one foot directly in front of the other with their eyes closed for 10 seconds.

Walking

11. Persons will be asked to walk with their current gait aid to where they can without a rest. Testing ceases if the person stops to rest. The person uses the gait aid that is currently most appropriate for them. If either of two gait aids could be used, the aid that provides the person with the highest level of independence should be used. Testing ceases once the person reaches 50 meters.
12. Independence is assessed over the person's maximum walking distance up to 50m (from item 11).

Dynamic Balance

13. A pen is placed 5 cm in front of the person's feet in standing. The person is asked if they can pick the pen up off the floor.
14. Walks backwards 4 steps. Person remains steady throughout.
15. Person can jump. Both feet clear the ground. Person remains steady throughout.

Definitions

Minimal assistance = "hands on" physical but minimal assistance, primarily to guide movement.
Supervision = another person monitors the activity without providing hands on assistance. May include verbal prompting.
Independent = the presence of another person is not considered necessary for safe mobility.

PROTOCOL FOR ADMINISTRATION OF THE DEMMI

1. Testing should be performed at the person's bedside.
2. Testing should be performed when the person has adequate medication eg. at least half an hour after pain or Parkinson's Disease medication.
3. The test should be administered in the sequence described in sections A-E: bed transfers, chair transfers, static balance, walking and dynamic balance.
4. Each item should be explained and, if necessary, demonstrated to the person.
5. Items should be ticked to indicate item success or failure. Reasons for not testing items should be recorded.
6. Items should not be tested if either the test administrator or the person performing the test are reluctant to attempt the item.
7. Persons should be scored based on their first attempt.
8. If an item is not appropriate given a person's medical condition, the item should not be tested and the reason recorded.
9. Persons can be encouraged but feedback should not be provided regarding performance.
10. Three equipment items are required: chair with 45cm seat height with arm rests, a hospital bed or plinth and a pen.
11. The person administering the test manipulates person medical equipment during testing (eg. portable oxygen, drips, drains etc) unless the person requires minimal assistance to perform the test and then a 2nd person will be required to assist with medical equipment.
12. For persons that require a rest after each item due to shortness of breath, a 10 minute rest should be provided half way through testing i.e. after completing the chair transfers section.
13. For person's who have low level mobility and require a hoist to transfer in/out of bed or chair, the chair section can be administered before the bed section for these persons.
14. Bed transfers: the bed height should be appropriate for the individual person. A standardised hospital bed or plinth should be used for testing. The person cannot use an external device such as the monkey bar, bed rail, edge of bed or a bed pole. Additional pillows may be provided for persons who are unable to lie flat in supine.
15. Chair transfers: A standardised chair height of 45cm is required. A firm chair with arms should be used.
16. Balance: Shoes cannot be worn for balance testing. The person cannot use external support to successfully complete any balance items. For sitting balance, neither the arm rests or the back of the chair can be used for external support. Standing balance tests should be performed with the person positioned between an elevated bed on one side and the test administrator on the other side. If a person displays unsteadiness or significant sway during testing, testing of that item should cease.
17. Walking: Appropriate shoes can be worn for walking tests. The same shoes must be worn for repeat testing.
18. Scoring: Using the conversion table provided, the raw score total must be converted to a DEMMI SCORE.

© Copyright de Morton, Davidson & Keating 2007. The DEMMI may be printed or reproduced without alteration (retaining this copyright notice). All other rights reserved. For other authorisations (including to translate the DEMMI) contact Dr Natalie de Morton: natalie.demorton@med.monash.edu.au

The development of the DEMMI has been supported by a post graduate scholarship from the National Health and Medical Research Council of Australia (Dora Lush Postgraduate Scholarship, Grant no. 280632), funded by the HCF Health and Medical Research Foundation and also supported by The Northern Clinical Research Centre, Northern Health.

The DEMMI should be cited as: de Morton NA, Davidson M, Keating JL. The de Morton Mobility Index (DEMMI): an essential health index for an ageing world. *Health and Quality of Life Outcomes* 2008, 6:63.
